# Pesticide data program: 30 years of food residue data and trends

**DOI:** 10.1038/s41370-022-00482-1

**Published:** 2022-10-30

**Authors:** Chris Pappas, Brenda Foos

**Affiliations:** 1 Retired, United States Department of Agriculture (USDA), Washington, DC USA; 2 USDA Agricultural Marketing Service, Science and Technology Program, Monitoring Programs Division, 1400 Independence Avenue, SW, Washington, DC 20250 USA

**Keywords:** Pesticide Data Program, PDP, Pesticide residues, Food safety, Food monitoring

## Abstract

The USDA’s Pesticide Data Program (PDP) celebrated its 30th anniversary in 2021 and is one of the world’s largest monitoring programs for pesticide residues. The PDP database contains over 42 million data points for a pesticide paired to a commodity that have resulted from the analysis of nearly 310,000 food samples of 126 different commodities. Over the decades of the program, sampling methods and infrastructure, major milestones, developments, and accomplishments have unfolded. Comparisons of data for four commodities that were in the program early on illustrate that over time pesticide residues on foods change, particularly when new pesticides are registered, and updated data, such as those provided by PDP, are key for exposure and risk assessment.

## Introduction

The Pesticide Data Program (PDP) is the United States Department of Agriculture’s (USDA’s) nationally representative monitoring program for pesticide residues in the U.S. food supply. PDP began operations in 1991, and 2021 marked the 30-year anniversary of the program. The program is a Federal-State partnership to collect and test foods that are available to the American consumer. The current PDP database contains 42 million residue data points that pair an individual pesticide residue with a food commodity. The data are used by the government, industry, researchers, and the public for assessing dietary exposure and risk, marketing U.S. commodities, and showing pesticide residues at the consumer level are generally below established limits. The limits for the maximum amount of a pesticide allowed to remain in or on food are known as Maximum Residue Levels (MRLs) internationally and as tolerances in the United States [1].

In February of 1989, the popular television program 60 min aired the segment, “A is for Alar” [2] based on a Natural Resources Defense Council (NRDC) report titled “Intolerable Risk: Pesticides in Our Children’s Foods” [3]. Subsequently, the President’s 1989 Food Safety Plan called for streamlining the ability to remove potentially hazardous pesticides from the market, and in response, USDA established PDP [4]. Within USDA, the Agricultural Marketing Service was tasked to provide objective, comprehensive data on actual pesticide residues in food at the consumer level [4]. The Food Quality Protection Act of 1996 authorized the program and added an emphasis on the foods most likely consumed by infants and children [5].

The PDP framework was developed in 1990 and 1991 to collect samples close to the consumer to reflect realistic exposures. The key concepts included a statistically based sampling plan, a rigorous quality assurance/quality control (QA/QC) program, standardized protocols for sampling and testing, electronic reporting of data, state-of-the-art analytical capacity, and voluntary participation. The focus was to collect food commodities that are highly consumed by the American public [6]. Today, standardized procedures are used for the collection, receipt, processing, testing, and reporting of PDP samples. The standardized operating procedures (SOPs) are discussed in more detail in the following sections and are publicly available [7].

PDP was developed as a voluntary program that works with State agencies covering all four census regions and nearly one-half of the U.S. population [8]. Current PDP participants include California (CA), Colorado (CO), Florida (FL), Maryland (MD), Michigan (MI), New York (NY), Ohio (OH), Texas (TX), and Washington (WA). States that previously have been PDP participants include Minnesota (MN), Montana (MT), North Carolina (NC), and Wisconsin (WI).

PDP works with Federal Agencies, including the Environmental Protection Agency (EPA) and Food and Drug Administration (FDA), on the selection of commodities and pesticides tested, along with these other USDA partners on the collection and utilization of PDP data: National Agricultural Statistics Service (NASS), Office of Pest Management and Policy (OPMP), and the Foreign Agricultural Service (FAS).

A review of PDP operations and findings across 30 years of results has not previously been published. PDP data are publicly available and searchable [9].

## PDP sampling methods and infrastructure

The goal of PDP’s sampling operation is to obtain a valid representation of the U.S. food supply. Without a statistically based sampling frame, the data generated by the PDP testing laboratories would not be fit for their intended uses in exposure and risk assessment. NASS developed a sampling frame to meet the intended purposes and the ensuing publication, *Developing an Estimation Strategy for a Pesticide Data Program* [10], provided that framework.

PDP sampling uses a probability-proportional-to-size model [10] wherein each State assigns every participating site a volume indicator compared to other sites in the State. The volume indicator is used to ensure bigger sites are selected more often. Volume indicators run from 1 to 10 and a site with a volume of 5 would be five times more likely to be selected than one with a volume indicator of 1.

Another important part of the sampling framework is randomness in both site selection and sample selection. Random site selection is performed prior to the calendar quarter, and each State has established random sample selection procedures for staff visiting the collection sites. The combination of probability-proportional-to-size with random site and sample selection was established to give each pound of product an equal chance of selection [10]. Each participating collection State works with NASS to develop procedures for site weighting and selection.

Other key concepts of representative sample collection include having the population of participating States total close to 50% of the U.S. population and covering all four U.S. Census regions [11]. Further, the population of each participating collection State is used to apportion the number of scheduled samples within the program. In 2021 [12], nine States collected 59 samples per month for each PDP commodity. The breakdown is as follows: California – 13 samples, Colorado – 2 samples, Florida – 7 samples, Maryland – 4 samples, Michigan – 6 samples, New York – 9 samples, Ohio – 6 samples, Texas – 8 samples, and Washington – 4 samples. Previously, North Carolina (1993–1996, 2011–2020) and Wisconsin (1996–2013) were also sample collection States.

PDP rotates commodities from year to year in order to include more commodities in the program. Fresh commodities typically remain in the program for two years to capture annual and seasonal variation, while processed commodities typically are sampled for one year. High-consumption commodities are rotated through the program approximately every five years to capture any changes in agricultural practices and to ensure fresh monitoring data are available for dietary risk assessments. Other commodities are included in the program when EPA expresses an interest in the data, and commodities chosen for inclusion or rotation in PDP are based on EPA data needs.

Fruit, vegetable, nut (almonds and peanut butter), dairy (butter, heavy cream, and milk), egg, honey, barley, oats, rice, wheat flour, catfish, salmon, and bottled water samples are collected by trained State personnel at terminal markets, distribution centers, and other wholesalers. This allows samples to be collected close to the point of consumption to better estimate exposure at the consumer level. Samples are randomly chosen without regard to country of origin, variety, or their status as organic or conventional. PDP issues fact sheets for each commodity that specify acceptable and unacceptable products, target sample size (typically 3–5 lb., 1.4–2.3 kg), instructions for completing the electronic sample information form (e-SIF), along with packing and shipping details.

The e-SIF is used to capture information regarding the sample. A unique sample ID is comprised of the collection State, year, month, date, site code, commodity code, and laboratory code. Other data are captured as available and include country of origin, variety, product claim (e.g., organic), commodity type (e.g., fresh, frozen, dried, etc.), package/container type (e.g., plastic clamshell, cardboard box, etc.), and information on the type of facility (e.g., distribution center, terminal market, etc.).

Once samples are collected and sample information recorded, they are packed and shipped to the testing laboratory on the same day. Program-specific requirements that sample collectors across all States adhere to are outlined in the PDP-issued Sampling SOP [13].

Special collection approaches have been used for select commodities. Whole grain corn, soybean, and wheat samples were collected by trained USDA Federal Grain Inspection Service (FGIS) inspectors. Beef (adipose, liver, and muscle), pork (adipose and muscle), and poultry (adipose, liver, muscle-breast and thigh) samples were collected by trained USDA Food Safety and Inspection Service (FSIS) inspectors. Treated and untreated drinking water samples were collected onsite by trained personnel at selected water treatment facilities across the country, and potable groundwater samples were collected from private domestic wells by homeowners and school/childcare facility personnel.

## Major milestones, developments, and accomplishments

Over its 30-year history, PDP has generated close to 42 million pesticide/commodity data points from the analysis of almost 311,000 samples over 126 different commodities. That’s a long way from the start of the program in May 1991 when three States (FL, NY, and WA) collected three commodities (grapes, lettuce, and potatoes) for a total of 35 samples per month.

In 1991, samples were analyzed for 11 pesticides and only detected values were reported. In 2021, nine States collected 14 commodities each month for a total of 826 samples per month. Samples are analyzed for up to 515 residues, and both detects and non-detects are reported.

**Fig. 1:**
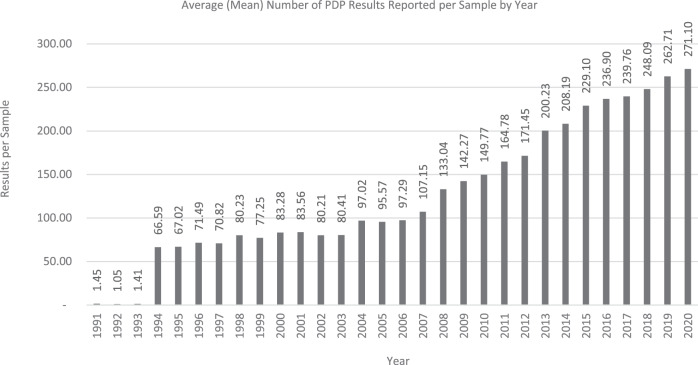
Mean results per sample. Average (mean) number of PDP results reported per sample by year.

Reporting non-detects was an early major program development. For the years 1991–1993, the testing laboratories only reported data for the confirmed detection of pesticide residue. Beginning in 1994, testing laboratories began reporting all validated pesticide residues results including non-detects which is key for application of monitoring data in exposure assessment. The number of reported pesticide/commodity data points increased significantly. In 1993, the analysis of 7328 samples yielded 10,329 discrete pesticide/commodity data points (1.41 data points per sample). In 1994 with non-detects reported, 7,589 samples yielded 504,296 discrete pesticide/commodity data points (66.6 data points per sample). In 2020, the analysis of 9600 samples yielded 2,602,551 discrete pesticide/commodity data points (271.1 data points per sample; Fig. 1).

Trans-shipping was a major program development that affected both sampling and testing. Prior to trans-shipping, the testing laboratory would analyze the samples for all commodities collected in their State. Because different commodities could not be combined into a single analytical set, the testing laboratories had to run smaller sets including required quality assurance samples with each set. This prevented economy-of-scale and reduced productivity. Additionally, switching between the different commodities had the potential to impact proficiency as matrix effects vary dramatically (e.g., testing grapes is very different than spinach). In October 1994, a pilot project between three States (CO, MI, and WA) began to send samples from all the States to a single testing laboratory, thereby allowing for the creation of larger sample sets. By 1997, all States were trans-shipping samples. This increased proficiency and productivity while also substantially reducing mandatory method validation and quality assurance costs.

Throughout the entire history of PDP, there has been a continued drive to lower detection limits and increase the number of pesticide residues included in the screens. For example, in 1996 the mean limit of detection for thiabendazole was 53.5 parts-per-billion (ppb) while in 2020 the mean limit of detection was 3.9 ppb. Simultaneously, there has been an increase in the number of compounds included in the screens (Fig. 1). Because PDP data are geared for dietary risk assessments, the current laboratory methods are optimized for detection limits in the low ppb.

### Laboratory analysis

The multi-residue analytical methods employed at PDP laboratories have evolved over the years. While both gas and liquid chromatography instruments have been used throughout the entirety of the program, the detectors coupled to those have changed considerably. Initially, determinations by gas chromatography included the use of electron capture detector (ECD), flame photometric detector (FPD), electrolytic conductivity detector (ELCD), and flame ionization detector (FID) along with mass spectrometry (MS) and liquid chromatography determinations primarily used post column derivatization and ultraviolet detectors. Gradually, the laboratories made the switch to single-stage MS or tandem mass spectrometry (MS/MS) and by 2012 all testing laboratories had made the move to all MS confirmation.

The extraction methods used by the PDP laboratories have evolved along with the analytical methods. Variations of the Luke [14] extraction, a solid phase extraction based on the Agriculture and Agri-Food Canada method [15], and a multi-residue extraction method developed by the California Department of Food and Agriculture (CDFA) [16] were initially used by the PDP laboratories. Currently, all use variations of the QuEChERS [17] method. Procedures for inspecting, storing, and processing samples are specified in the PDP LABOP SOP [18]. Upon receipt, samples are inspected for acceptability and damage and discarded if deemed inedible. Samples can be stored for specified periods of time and temperature prior to preparation. Samples are processed to mimic consumer practices: the sample is rinsed, ends are trimmed, and inedible portions (e.g., stems seeds, peels, rinds) are removed. The prepared food samples are then chopped, blended, or mixed until homogenized. Homogenates are either extracted immediately or held at −40 °C until extraction.

Just as the extraction and analytical methods have evolved over the years, so have the program SOPs [7]. During 2009–10, PDP merged the requirements of 48 SOPs into four overarching documents: administration, laboratory operations, data reporting, and QA/QC. This consolidation removed reduncancies and inconsistencies. In 2016, the requirements from nine sampling SOPs were consolidated into a single document.

### Special projects

PDP has conducted many special projects during the past 30 years. From 1997 through 2000, PDP conducted surveys of single servings of apples, peaches, pears, and potatoes. Surveys of single servings are designed for commodities in which one unit may comprise a serving for a single meal/snack. In surveys of single servings, the samples are processed differently than routine PDP composite samples. For routine PDP samples, the entire 5 lb. sample is homogenized into a composite sample, whereas, for surveys of single servings, a single unit (e.g., an apple) is taken from the 5 lb. sample and homogenized. Details regarding sample collection, preparation, analysis and results can be found in the respective PDP Annual Summaries [19].

PDP conducted a special triazoles survey in 2003–2004 [20]. EPA requested data for triazole pesticides and their common metabolites as part of a review of new tolerance applications. PDP partnered with the United States Triazole Task Force (USTTF) to test apples, bananas, eggs, grapes, milk, peaches, peanut butter, strawberries, and wheat. Sample analysis was split between PDP laboratories and USTTF contract laboratories.

In 2005, PDP responded to a data request from EPA to collect 306 soybean samples slated for export and test for 14 fungicides used to treat soybean rust and two insecticides used to control the Chinese aphid [21]. The USDA Grain Inspection, Packers, and Stockyard Administration (GIPSA) laboratory developed a specialized method to optimize the recovery of target compounds using a solid phase extraction and liquid chromatography/MS/MS instrumentation.

In 2009, PDP conducted a special survey where 387 organic lettuce samples [20, 22] were collected at routine PDP sampling sites. The samples were tested for 57 parent pesticides, metabolites, and isomers with an emphasis on compounds used in organic farming.

In 1998 and 1999, PDP teamed with the Corn Refiners Association to test corn syrup. In 1998, 298 one-quart high fructose corn sugar samples were collected by plant personnel at one of the 17 participating plants that represented over 95% of corn syrup production. The samples were sent to a PDP laboratory and the testing profile included 83 pesticides and 26 metabolites, degrades, or isomers [23]. In 1999, a total of 156 dextrose equivalent corn syrup samples were collected by plant personnel at one of the eight participating refineries. The samples were sent to a PDP laboratory and the testing profile included 82 pesticides and 21 metabolites, degrades, or isomers [24].

Details for all PDP special projects can be found by visiting the “Special Projects” tab of the PDP website [20] and details on the results of the special projects and compounds detected can be found in the corresponding *PDP Annual Summary* reports [19].

## Data analysis and trends

With almost 42 million pesticide-commodity data points in the PDP database, the options for data mining are endless. The reader is encouraged to explore the PDP searchable database [9] or utilize downloadable annual data files at www.ams.usda.gov/pdp. As examples of commodities with data from the early years of the program, the results from apples, carrots, grapes, and green beans from 1994 are compared to the more recent results for these same commodities. Prior to 1994, PDP laboratories only reported positive detections, so the years 1991–1993 were excluded from the examples.

**Fig. 2:**
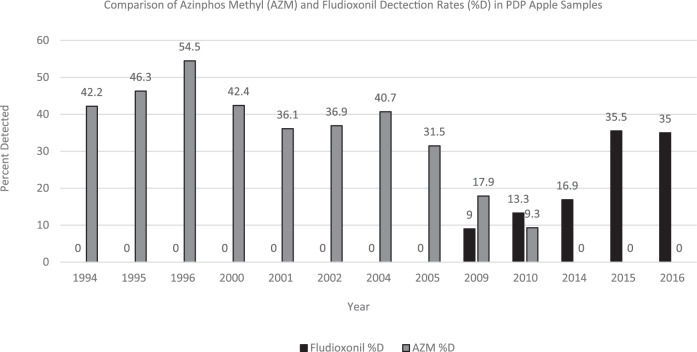
Detection rates of select pesticides in apples. Comparison of azinphos methyl (AZM) and fludioxonil detection rates (%D) in PDP apple samples.

The 1994 apple data [25] show the five most frequently detected residues were diphenylamine, thiabendazole, azinphos methyl, propargite, and carbaryl. There were 86 compounds included in the testing screen and 29 were detected. By 2016 [26], the most recent PDP analysis of apples, both testing and agricultural practices had changed. There were 201 compounds included in the testing screen and 47 were detected. Diphenylamine and thiabendazole were still the two most frequently detected residues in 2016; however, the rest of the top five (fludioxonil, pyrimethanil, and acetamiprid) were not yet registered for use in 1994. Conversely, two of the top five in 1994 (azinphos methyl and propargite) were not detected in any of the 2016 apple samples. This can be attributed to the cancellation of all crop uses of azinphos methyl in 2012 [27] and propargite use on apples in 1996 [28]. Carbaryl was detected in less than 1% of the 2016 apple samples, far below its 1994 level of 21%. PDP data suggest that fludioxonil replaced azinphos methyl as the third most commonly detected residue in apples (Fig. 2).

**Fig. 3:**
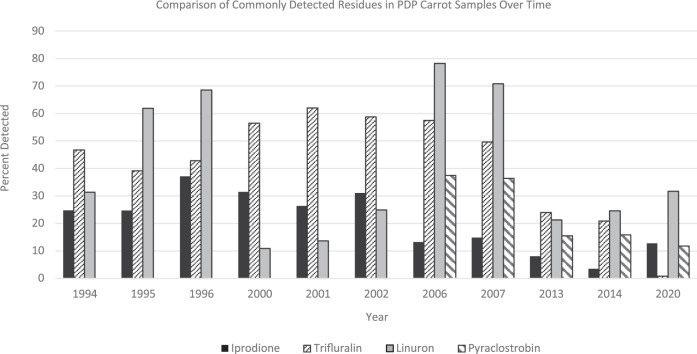
Detection rates of select pesticides in carrots. Comparison of commonly detected residues in PDP carrot samples over time.

The 1994 carrot testing screen included 85 compounds and 22 were detected. By 2020 [29], the testing screen included 327 compounds and 28 were detected, though the most frequently detected compounds had changed (Fig. 3). In 1994 [25], the five most frequently detected residues were DDE, trifluralin, iprodione, diazinon, and linuron. In 2020 [29], the five most frequently detected residues were linuron, boscalid, iprodione, pyraclostrobin, and penthiopyrad. Three of the top five detected residues in 2020 (boscalid, pyraclostrobin, and penthiopyrad) were not registered for use on carrots in 1994 and were not included in the screen. In 1994, trifluralin was detected in 46.7% of the carrot samples, while in 2020 it was detected in less than one percent. This can most likely be attributed to the emergence of new compounds as there still is an active trifluralin tolerance for carrots.

**Fig. 4:**
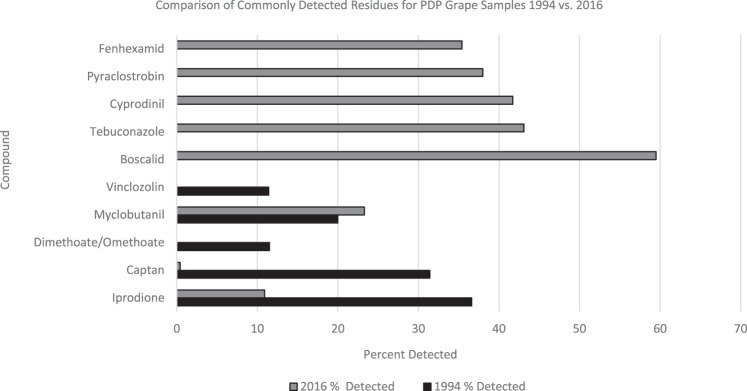
Detection rates for select pesticides in grapes. Comparison of commonly detected residues for PDP grape samples 1994 vs. 2016.

The 1994 grape testing screen included 85 compounds and 28 were detected. In 2016, the testing screen included 167 compounds, 56 of which were detected. In 1994, there were six compounds detected in more than 10% of the grape samples. By 2016, three of the six were not detected in any samples due to the cancellation of their use and a fourth was detected in only three of the 708 grape samples tested. The three 1994 residues not detected in 2016 include dimethoate, omethoate, and vinclozolin (Fig. 4). The use of dimethoate on grapes was cancelled in 2004 [30] (thereby impacting its metabolite omethoate) and vinclozolin use on grapes was cancelled in 1997 [31]. In 1994, captan was detected at a significantly higher rate (31%) than in 2016 (0.4%). All top five most frequently detected grape compounds in 2020 were not registered for use in 1994: Boscalid was first registered in 2003 [32], tebuconazole [33] in 1999, cyprodinil [34] in 1998, pyraclostrobin [35] in 2002, and fenhexamid [36] in 1999.

**Fig. 5:**
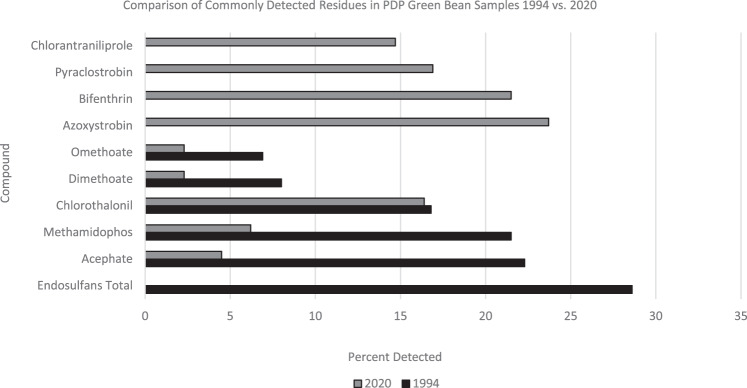
Detection rates for select pesticides in green beans. Comparison of commonly detected residues in PDP green bean samples 1994 vs. 2020.

The 1994 green bean testing screen included 84 compounds and 26 were detected. In 2020, the testing screen included 524 compounds, 61 of which were detected. In 1994, endosulfan was the most frequently detected residue in green beans while in 2020, it was not detected in any samples. This reflects the cancellation of its use on green beans in 2010 [37]. In 1994, the top five most detected residues were endosulfan, acephate, methamidophos, chlorothalonil, and benomyl (Fig. 5). In 2020, the five most frequently detected residues were: azoxystrobin, carbendazim, bifenthrin, pyraclostrobin, and chlorothalonil. The 2020 residue detected most frequently in green beans, azoxystrobin [38], was not registered for use in 1994. Detection rates for acephate and its metabolite, methamidophos, went from 22.3% and 21.5%, respectively, in 1994 to 4.5% and 6.2% in 2020. The detection rate for chlorothalonil was one of the most consistent across time: 16.8% in 1994 and 16.4% in 2020.

**Fig. 6:**
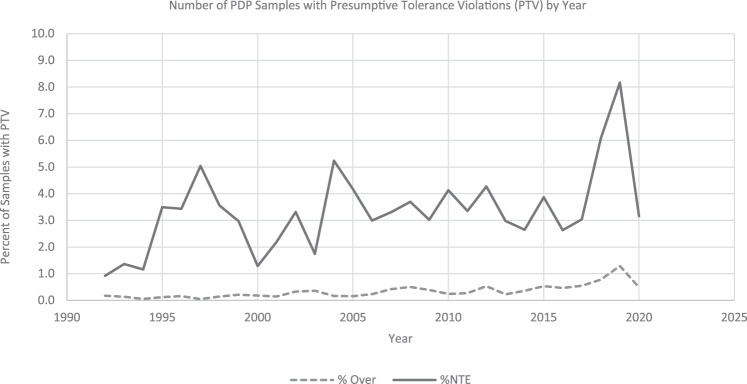
Number of PDP samples with presumptive tolerance violations by year. The dashed line indicates the number of PDP sample with residues exceeding the established tolerance, and the solid line indicates the number of residues detected that did not have an established tolerance.

PDP evaluates whether monitored residues exceed existing tolerances. When they do, the results are reported to EPA and FDA as presumptive tolerance violations (PTV) and are included in the *PDP Annual Summary* [19] report. PDP data show that when pesticide residues are found on foods, they are nearly always below the maximum levels that are established by the EPA and enforced by the FDA (Fig. 6). Besides the residues exceeding tolerances, PDP also monitors another type of PTV. When a residue that does not have an active tolerance on a commodity is detected at any level, it is a no tolerance established (NTE) PTV. The rate of PTVs in a particular year is largely dependent on the foods being tested as PDP rotates commodities, and some have been found to have higher rates of PTVs than others.

## Data use and impact

PDP data’s impact results from the use of the data in exposure assessment, and EPA is a primary user of PDP data. Pesticides prioritized for screening by PDP include those with currently registered uses for the commodity being tested and compounds for which toxicity data and preliminary estimates of dietary exposure indicate the need for more extensive/updated residue data. PDP data are used by EPA to refine dietary exposure assessments during the review of the safety of existing pesticide tolerances, including assessments completed as a part of the pesticide registration review process under the Food Quality Protection Act of 1996. The EPA’s acute dietary exposure and risk assessment for cyfluthrin can be used as a case study of the use of PDP data in an EPA risk assessment for pesticide registration review [39]. In this assessment, PDP data were used in the refined acute probabilistic dietary exposure analysis for most commodities. In conjunction with EPA’s exposure assessment efforts, PDP monitoring data are also incorporated into the World Health Organization’s (WHO) Global Environment Monitoring System - Food Contamination Monitoring and Assessment Programme (GEMS/Food), a data platform used by the Joint Food and Agriculture Organization of the United Nations (FAO)/WHO Meeting on Pesticide Residues to evaluate dietary exposure and recommend the establishment of pesticide maximum residue limits (MRLs) to the Codex Committee on Pesticide Residues [40].

PDP data are also used by EPA to develop the Report on the Environment [41] and by FDA to assist in planning commodity surveys for pesticide residues as a part of the Agency’s enforcement and regulatory programs. Additionally, the program has had impact in the international community through consultation with foreign efforts to develop monitoring programs that parallel PDP.

Because PDP data are downloadable in their entirety, the data have been utilized in many research efforts and as a result have been included in numerous peer-reviewed scientific journal articles. The data are also utilized by various industry, environmental and media organizations in their independent publications.

The current uses of PDP data illustrate that the data have impactful application in exposure science. Given the large size of the PDP database, there are likely many more opportunities for data analyses and applications in exposure assessments that have yet to be explored.

## Looking toward the future

In 1991, no one could have predicted that 30 years later, there would be 42 million pesticide-commodity data points in the PDP database. Even as we look back, we are looking for ways to improve the program so that it can continue to be a valuable resource into the future. Increasing commodities tested, incorporating more modern extraction and detection equipment, and adding more states and testing laboratories to the PDP would bring about positive changes. PDP has never been a static program, and change has been a constant.

Expansion of testing profiles has been a program hallmark since PDP started. Once PDP began reporting non-detects as well as detects, the number of results per sample has steadily increased (Fig. 1). In the 12-year period of 2008–2020, the mean number of results per sample has more than doubled, going from 133 to 271. The PDP laboratories have responded to the challenge of increasing their multi-residue screens and this trend is expected to continue.

Over the course of its history, PDP has tested 126 different commodities from apples to zucchini [42], and with greater resources, it could include new commodities from arugula to yucca. Adding new commodities expands the information for dietary exposure and risk assessment and allows for additional comparison of actual monitoring data with modeled data.

Sampling different foods, as well as fresh and processed products, illustrates residue differences within and between commodities. For example, when PDP allows frozen as well as fresh commodities, an extra benefit is that frozen samples from countries that do not typically export fresh product to the USA are available for collection.

However, funding is typically prioritized for refreshing data on existing, high consumption commodities. With limited and shrinking resources, PDP rotates commodities out of the program after one or two years of collection. Commodity changes are made quarterly through consultation with EPA regarding its data needs, and typically up to four commodities rotate at each interval. As such, the program cannot easily expand to include new commodities, nor consistently test all commodities every year.

In addition to expanding commodities for testing, expanding the residues that can be tested will allow the program to continue improving. With more resources, PDP could expand new technologies for instrumentation and extraction to remain relevant in residue reporting. PDP went from gas chromatography and liquid chromatography instruments coupled to detectors for specific classes of compounds (e.g., organochlorines, nitrogen/phosphorous, etc.) in the early years of the program to all laboratories using tandem mass spectrometry for the past nine years. This shift allowed the laboratories to expand their testing screens and lower detection limits. As zero noise instruments, high resolution mass spectrometry, and other new instrumentation and data processing abilities emerge and improve, this trend of analyzing more compounds in each screen with better quantification limits is expected to continue, and PDP could improve with them [43].

Likewise, on extraction, PDP laboratories have progressed from the Luke extraction to the QuEChERS, and as a result have reduced the volume of solvents and waste generated over a wide range of commodity types. PDP will continue to explore cost-efficient new technologies to add new classes of compounds not currently amenable to multi-residue methods (e.g., macrocyclic lactones, acid herbicides, ethylene bisdithiocarbamate (EBDC) fungicides, etc.).

Finally, the addition of new collection States and/or testing laboratories would increase PDP’s capacity and output, qualitatively improving the representativeness of PDP data. To address representation within the program, PDP monitors population shifts in the U.S., and a reassignment of sample collection numbers based on these trends could be made after consulting with NASS and program partners. Collaborating with new states, in addition to the potentially expanding the commodities tested and updating laboratory approaches, will carry PDP into the future.

## Conclusions

Three decades of PDP data illustrate that pesticide residues are quantifiable for a wide variety of U.S. food commodities, and the residues are typically in compliance with the tolerance levels set by EPA (Fig. 6). The high-quality, nationally representative pesticide residue data provided by PDP are not only used in exposure and risk analyses undertaken by government, industry and research organizations; the data also contribute to the information available to help ensure consumer confidence in the foods they provide to their families.

There are extensive opportunities to analyze changes in pesticide residues in food commodities over time by using the 30 years and 42 million pesticide-commodity paired data points available through the PDP database. The trends seen in our examples support the conclusion that over time pesticide residues on foods change, particularly when new pesticides are registered and when updated data are needed for exposure and risk assessment. Residue monitoring and the rotation of food commodities by PDP will continue to be a valuable source of such information for these assessments.

Over 30 years, PDP has developed a comprehensive approach to pesticide residue monitoring in U.S. food commodities. The PDP database is currently one of the largest and longest-running sources of pesticide residues in foods globally and is used by Government, industry, and academia in a variety of applications. Entering the program’s 31st year, as PDP continues with technological advances, the database will provide even more robust data for pesticide residues and exposure analysis.

### Supplementary information

Reporting Checklist

## Data Availability

PDP Annual Summaries and database are available on the PDP website: https://www.ams.usda.gov/datasets/pdp/pdpdata.
